# How to Travel Cheaply

**Published:** 1882-03

**Authors:** 


### HOW TO TRAVEL CHEAPLY.

A writer in the New York *Evening Post* says: The secret of traveling in Europe, especially in Great Britain, is to do as one would at home. A person of moderate means on pleasure bent, but, like Mrs. Gilpin, 'of a frugal mind,' will soon perceive that he need not patronize the Langham Hotel to have a good time in London, or pay a guinea for a seat at the opera. As he is content at a boarding house at college or in New York,or an economical luncheon when on a journey in his own country, so in England or on the continent he will find, if he seeks them, pleasant and suitable accommodations at a reasonable price. If in London, for instance, he will find the thousands of rooms in the little streets running down to the Thames from the Strand, in the neighborhood of the British museum, or other pleasant localities, for $5 or $6 a week for lodging, attendance and breakfast. His other meals need not cost more than a $1 a day, thus leaving him several dollars for pocket money out of a weekly allowance of even $20. Conveyances are very cheap and most of the best "sights" are either free or to be seen for a trifling charge. What I have said of London applies with equal force to Paris, Berlin, or any of the great cities. But for a foreigner readily to avail himself of these advantages on the continent he should have a familiar acquaintance with the language of the country. Next to the great item of steamer fares, the most depleting and afflicting pecuniary items will be connected with inland travel, and if your means are limited you will get much more satisfaction from a trip by studying a few of the best places thoroughly rather than rushing from one place to another and catching only casual glimpses of everything seen. One can make a route estimate at home of what travel in England will cost, and thus, in a measure, determine what excursions are practicable by taking a serviceable map and measuring distances. Railroad fares in Great Britain are pretty uniformly two, three and four cents a mile, according to class of carriage. With respect to these classes, the bulk of the travel is by the third-class, and second-class is good enough for any one. The English have a saying that only fools and Americans travel first-class.

---

GREAT men undertake great things because they are great, and fools because they think them easy.

DON'T WORRY THE BEES.—The stings of bees were given them for the protection of their stores. They are not disposed to sting when not in danger, and every bee which does sting dies. Away from their own hive they rarely make an attack. The natural dread of stings deters many from keeping bees who would be glad to do so. In the use of modern hives the danger of being stung is lessened, as these give you facilities for subduing them. A bee with its honey sac full never stings. When you alarm a colony of bees, they all instinctively at once fill their sacs with honey, and after time has been allowed them to do this, the hive can be opened and examined with no danger from their anger.

PEPSIN is a nitrogenous substance existing in the gastric juice and as a viscid matter in the peptic gland and on the walls of the stomachs of animals. The mucous membrane of the stomach (of the hog, sheep or calf, killed fasting) is scraped, and macerated in cold water for twelve hours; the pepsin in the strained liquid is then precipitated by acetate of lead, the deposit washed once or twice by decantation, sulphureted hydrogen passed through the mixture of the deposit with a little water to remove the whole of the lead, and the filtered liquid evaporated to dryness at a temperature not exceeding 105 degrees Fah. As met with in pharmacy, the strength of pepsin varies greatly. It is often prepared by simply mixing with starch the thick liquid obtained on macerating the scraped stomach with water, and evaporating to dryness. The composition of pepsin is not positively known.

